# Recent Applications of Molecularly Imprinted Sol-Gel Methodology in Sample Preparation

**DOI:** 10.3390/molecules24162889

**Published:** 2019-08-09

**Authors:** Mohammad Mahdi Moein, Abbi Abdel-Rehim, Mohamed Abdel-Rehim

**Affiliations:** 1 Department of Radiopharmacy, Karolinska University Hospital, S-171 76 Stockholm, Sweden; 2 Faculty of Science and Engineering, University of Manchester, Manchester M13 9PL, UK; 3 Karolinska Institutet, Department of Clinical Neuroscience, Centre for Psychiatric Research, Karolinska Hospital, S-171 76 Stockholm, Sweden; 4 Functional Materials Group, Department of Applied Physics, School of Engineering Sciences, KTH Royal Institute of Technology, SE-164 40 Stockholm, Sweden

**Keywords:** molecularly imprinted polymers, sol-gel, solid phase extraction, solid phase microextraction, in-tip, monolithic column, nanofiber, magnetic nanoparticles, dummy

## Abstract

Due to their selectivity and chemical stability, molecularly imprinted polymers have attracted great interest in sample preparation. Imprinted polymers have been applied for the extraction and the enrichment of different sorts of trace analytes in biological and environmental samples before their analysis. Additionally, MIPs are utilized in various sample preparation techniques such as SPE, SPME, SBSE and MEPS. Nevertheless, molecularly imprinted polymers suffer from thermal (stable only up to 150 °C) and mechanical stability issues, improper porosity and poor capacity. The sol-gel methodology as a promising alternative to address these limitations allowing the production of sorbents with controlled porosity and higher surface area. Thus the combination of molecularly imprinted technology and sol-gel technology can create influential materials with high selectivity, high capacity and high thermal stability. This work aims to present an overview of molecularly imprinted sol-gel polymerization methods and their applications in analytical and bioanalytical fields.

## 1. Introduction

Molecularly imprinted polymers (MIPs) as a privileged sorbent provide selective recognition sites for a template molecule of interest based on its size, structure and functional groups. MIPs were synthesized by non-covalent methods and implemented for the first time in the 1970s by Mosbach and his group [1]. MIPs as capable materials with high specificity, and their physical and chemical stability have been effectively used in the extraction, and microextraction fields and even in sensing applications [2,3,4,5,6,7,8,9,10,11,12,13,14,15]. The common MIP preparation methods require organic monomers (acrylates or acrylic acid) and an organic solution phase which represent limitations from environmental and biological points of view. The common MIP preparation methods (bulk and precipitation) suffer from limitations, such as the short life span of prepared polymers and the necessity of a relatively expensive initiator (azobisisobutyronitrile—AIBN). Moreover, both covalent and non-covalent MIP preparation methods suffer from some drawbacks such as template leaching, both low thermally and chemically stability, and poor reusability [16]. To overcome these limitations the sol-gel method was proposed as a simple, relativity low-cost methodology providing products of high thermal and mechanical stability as solid phases for applications in various areas of research [17]. Sol–gel synthesis occurs by dissolving a metal oxide precursor (M(OR)_n_) in a low molecular weight solvent medium using a catalyst (acid, base, or ions as F^−^) followed by a hydrolysis (water) and polycondensation step. Sol-gel is a simple, manageable and cost-effective method for the production of homogeneous and highly porous metal oxide nanosorbents. The sol–gel process gives us a chance to produce various sort of nanomaterials or to modify polymer surfaces for applications in different sample preparation techniques [17]. Sorbent swelling, structure deformation and blockage are common problem with MIPs in biological samples. Sol-gel chemistry can produce imprinted selective cavities with longer lifetime due to the use of silica-based materials with strong and stable structures. In addition, efficient elimination of the template molecule from the MIP network after preparation has always been a challenging issue that can be significantly reduced with the sol-gel methodology. The high thermally stability silica materials allow the use of high temperatures to remove the template from the MIP network. The poor porosity and low surface area that are further drawbacks in MIPs can be improved by using silica-based precursors as high porous and capacious natural materials. 

**Figure 1:**
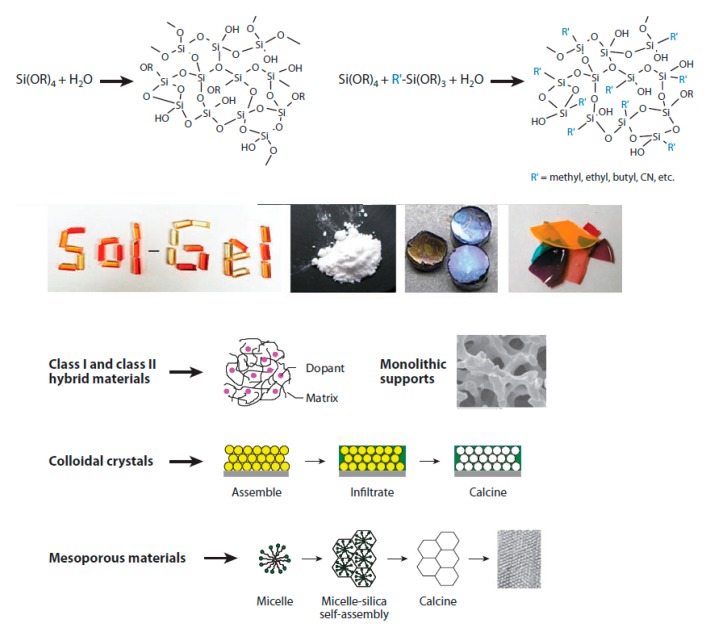
Sol-gel concept and variety [18].

The simple sol-gel technique concept and the various categories of possible sorbents accessible with this method has been classified by Collinson [18]. Briefly, numerous types of porous sorbents can be fabricated by performing polymerization, gelation, aging, drying, and heating as the main steps of the sol-gel method. By monitoring and optimizing the factors affecting each of the mentioned steps, different sorbent shapes such as uniform nanoparticles (1.5–10 nm), aerogels, xerogels, monoliths and thin films can be produced. In addition, the colloidal crystal templating method performed by implanting the silica into latex spheres has been presented to get pores in the range from 50 to 1000 nm diameter [19]. Sol-gel is a general way to create two groups of organic-inorganic substrates; hybrids of materials by weak interaction among organic and inorganic components (group I) or strong covalent binding with a siloxane matrix (group II) as shown in Figure 1 [18]. In a pioneering study by the Shea group, MIP were prepared by the sol-gel method for extraction of phosphates and phosphonates from aqueous samples and it was a great starting point for further developments in this field [20]. In this review we briefly cover the most recent MIP-sol-gel (MSG) preparation methods and their applications in extraction and microextraction studies.

## 2. MSG in Solid Phase Extraction (SPE)

SPE as the most common sample preparation technique, is the main beneficiary of MIP use [21]. However some aspects of the MIP-SPE method need to improve such as the low thermal stability, low adsorption capacity, short lifetime and low diffusion speed. The sol-gel technique has been modified and applied by many research groups to solve these limitations. In one study, MSG sorbent was used for on-line extraction and detection of enrofloxacin in fish and chicken samples [22]. In this work 3-aminopropyltriethoxylsilane (APTES) was employed as monomer, tetraethoxysilane (TEOS) as crosslinker and silica gel as support material in N,N dimethylformamide (DMF) solvent and suitable selectivity and sensitivity for measuring enrofloxacin was reported. Using relatively the same method MSG were synthesized and used for the extraction of methyl-3-quinoxaline-2-carboxylic acid and quinoxaline-2-carboxylic acid from pork muscle [23], cloxacilloic acid in cloxacillin [24], 2,4-dichlorophenoxyacetic acid [25], florfenicol [26], chrysoidine [27], vitamin D3 [28] and some polar organophosphorus pesticides from almond oil [29]. In an interesting method dummy-MSG coated with magnetic graphene oxide was synthesized for the extraction of phthalate esters from water and screening with GC/MS [30]. In a simple and cost-effective method a titania-based MIP was synthesized without monomer and crosslinker using sunset yellow (Sun) as template molecule due to high binding capacity between the Sun sulfonic acid groups with titanium under acidic conditions [31]. In a novel approach core-shell structural multi-walled carbon nanotubes (MWNTs)–Sudan IV MSG was prepared and used as SPE sorbent. The MWNTs–MIP was used for the on-line SPE–HPLC extraction and measurement of Sudan IV in chili samples [32]. 

MWNTs represent a remarkable support phase for making core-shell MIPs due to their significant strength, high surface area and unique chemical properties. The developed method showed high efficiency with over 89% recovery and 2.3 ng L^−1^ limit of detection. In this work 3-amino-propyltrimethoxysilane was used for covalent grafting of silicon–oxygen group onto the MWNTs’ surface. Then, a MIP was created on the MWNT surface using a template molecule, suitable monomer functional monomers and cross-linkers, followed by hydrolysis and condensation steps. Moreover, a magnetic surface ion-imprinted polymer (c-MMWCNTs-SiO_2_-IIP) was synthesized using magnetic CNTs/Fe_3_O_4_ composites (c-MMWCNTs) as the core, APTES as the functional monomer, TEOS as the cross-linker and applied for SPE of Cu(II) from herbal medicines [33]. In a one-step hydrothermal method, core–shell Fe_3_O_4_@MIP nanospheres were easily synthesized and applied for extraction and detection of bisphenol A in aqueous samples [34]. Also, MSG has been applied as SPE sorbent in food analysis and applied for the extraction of iprodione in a white wine sample [35]. MSG was prepared in various sorts and recently on a polyethylene support and used as µ-SPE and determination of methadone in human plasma [36]. Tablets was conditioned and immersed in plasma samples and the amount of extracted methadone was measured by LC-MS/MS. The referred study presented a simple method, however the lifetime and recovery aspects still need improvement. Moreover, diethyl-stilbestrol is a harmful residue to human health due to its potential carcinogenicity and in an important study it was extracted in milk samples by a magnetic MIP which was synthesized using a combination of bulk and sol-gel techniques [37]. The most problematic issue in MSG preparation is the leakage of template molecule, especially in the extraction of trace analytes in complex media. To overcome this problem the dummy silica MSG nanospheres method was presented by Liu for SPE of bisphenol A in food samples [38]. In the dummy method a compound with a similar structure to the desired analyte must be used as template molecule and in the mentioned study dihydroxybiphenyl was used as dummy template for bisphenol A MSG preparation. 

## 3. MSG in Solid Phase Microextraction (SPME)

**Figure 2:**
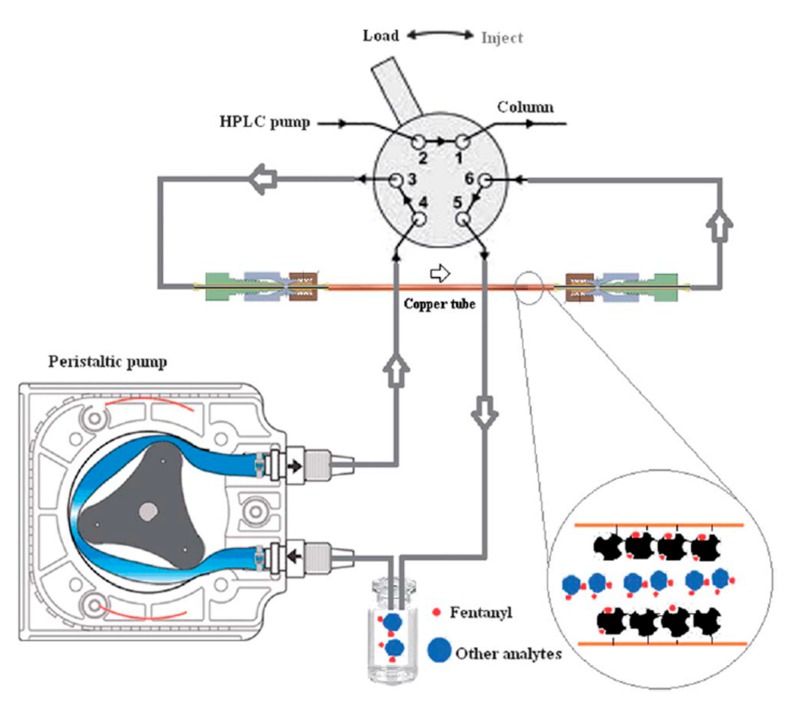
On-line capillary tube connected to HPLC [39].

The sol-gel method is suitable to solve some disadvantages of common MIP preparation methods such as swelling, low binding capacity and non-specific binding. Preventing the swelling and blockage is a crucial factor in the case of capillary sorbent preparation. In an intriguing study a molecularly imprinted xerogel (MIX) was used as a capillary sorbent for the microextraction of fentanyl from urine and plasma samples [39]. The xerogel was prepared by adding EPPTMOS (precursor) to fentanyl (template) using 10% (*v*/*v*) water and 70 µL TFA under sonication. A peristaltic pump was used to pass the prepared sol through a copper tube for 30 min to assure the formation of the gel on the inner surface of the copper tube. The tube was placed into a desiccator for up to 12–15 h for further aging and to increase polycondensation. The polycondensation step was accomplished by placing the loop in an oven in a temperature range of 50–200 °C for an appropriate period of time. Then, an organic solution (methanol and acetic acid (9:1)) was passed through the prepared loop to remove the template from the xerogel network. The prepared capillary tube was connected on-line to an HPLC loop (Figure 2) and used for all experiments. The developed on-line method showed recoveries of up to 85% for the extraction of fentanyl from biological samples. This robust and on-line method avoids protein precipitation and the dilution of plasma and urine samples.

**Figure 3:**
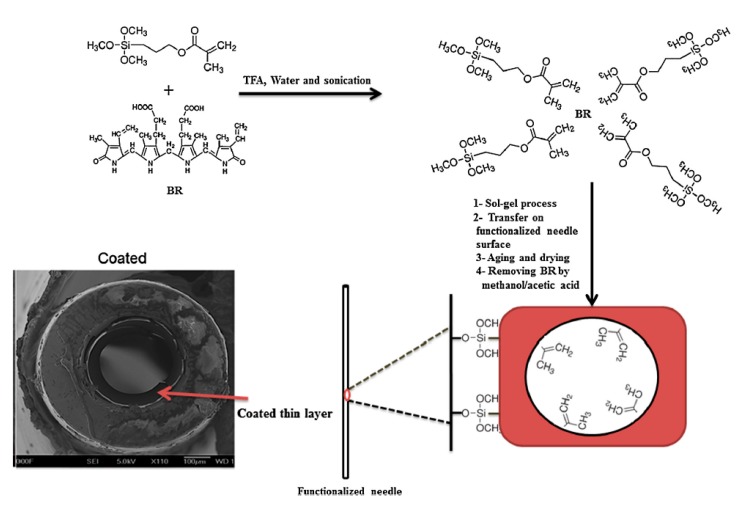
MIP xerogel in a needle [41].

In addition, MSG was used for the surface modification of a commercial fiber and applied for extraction of diazinon and its structural analogs from aqueous cucumber samples and detection with gas chromatography-nitrogen/phosphorus detection [40]. In another study the surface of a needle was optimized with a MSG xerogel method and used for the extraction of bilirubin (BR) from complex samples and screening with LC-MS/MS [41]. 3PMTMOS (precursor) and BR (template) were mixed and sonicated. Subsequently, TFA was added as catalyst and solution was sonicated, followed by the slow addition of water to begin the hydrolysis process and finally the prepared solution was incubated for 30 min at room temperature. Then, the mixture solution was passed through the needle to form a thin MSG surface and it was placed in a desiccator for 24 h to complete the aging process. Finally, the needle polycondensation process was accomplished in an oven using a temperature gradient between 50–250 °C for 3 h. This sorbent in comparison with a non-imprinted needle (as blank) showed roughly more than five-fold better imprinting factor, four times better recovery% and four times higher adsorption capacity. The prepared needle was connected to a Hamilton syringe and used for extraction purposes. This MSG showed stability and could be used for up to 100 extractions in complex biological solutions (Figure 3). This method can be a good recommended tool to solve the frangibility of solid phase fibers and can be applied in the SPME field. In addition, it is straightforward method and the product can be connected on-line to liquid and gas chromatography instruments. 

Moreover, in a further trend a SMPE-probe was prepared on the surface of a stainless-steel wire by the MSG method using chlorpyrifos as template molecule, tetraethoxysilane a sol-gel precursor, and acrylamide and β-cyclodextrin as functional monomers [42]. This probe was used for the residual determination of organophosphorous pesticides in fresh and dry foods by GC-FID. The MIP-SMPE-probe was a straightforward and robust tool, and showed good sensitivity, reproducibility and selectivity toward the investigated template molecules and their structural analogs.

## 4. Monolithic MSG

**Figure 4:**
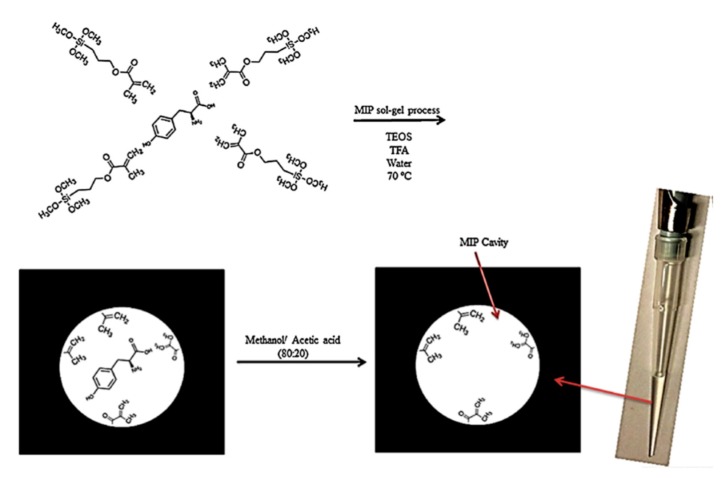
In-tip MSG [31].

High-throughput techniques like in-tip sample preparation methods have been considered due to their simplicity, stability and suitable recovery. In situ monolithic in-tip MIPs made by the sol-gel process are an easy, fast, robust and durable method that has been applied for the selective extraction of l-tyrosine (Tyr), a potential lung cancer biomarker, from biological fluids [43]. In this method the template molecule (Tyr) was mixed and sonicated with the precursor TPM for 30 min. Then, TEOS, TFA as the catalyst and water were added. The mixture was stirred for 30 min at 70 °C. After that, 0.03 mL of this solution was transferred into a tip, and the tip was kept in 70 °C for 2 h. Then, the tip was left at room temperature for 7 h. Finally, methanol containing 10% acetic acid was used as the solvent to remove the entrapped template (Figure 4). The in-tip monolithic MSG was used for Tyr extraction and measurement by LC-MS/MS with high recovery, accuracy and selectivity. Using almost the same process an in-tip dummy MIP for SPME of vanillin and methyl vanillin and their determination by HPLC was prepared [44].

## 5. Hollow Fiber and Nanofiber Modification and Preparation with MSG

Hollow fibers (HFs) are a well-known alternative to fragile SPME fibers due to their high stability and avoidance of biological matrix interference. The transfer speed of an analyte from solution to the surface is a key factor in HF performance which can be facilitated using solution stirring. Various modified sorts of HFs are mostly applied in the liquid-phase microextraction (LPME) field [45]. 

**Figure 5:**
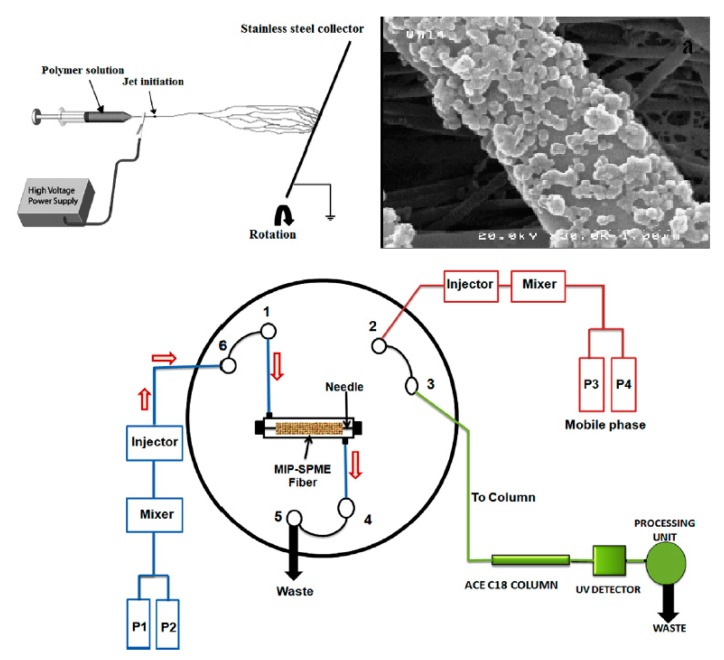
Schematic method using MIP nanofiber preparation, structure and operation [46].

Recently, the surface of a hollow fiber membrane was modified with MSG as LPME sorbent for the extraction of hippuric acid from human plasma and urine samples [45]. In this work a polysulfone HF membrane surface was modified with the sol-gel method and used for LPME extraction of hippuric acid from complex matrixes. In a further approach an electrospinning method was used for the preparation of the MSG nanofibers [46]. Electrospinning is a capable methodology to create micro-/nanofibers through an inexpensive and simple process. Electrospun micro-/nanofibers have been applied to many different applications. In on work, a simple and novel way for the preparation of unbreakable MSG nanofibers by the electrospinning technique was developed. The electrospinning of MSG is a challenging task to overcome this issue. Nylon 6 (12% *w*/*w*) in 4 mL formic acid was used as a backbone and support of the precursor (Figure 5). The developed method was used for SPME and determination of acesulfame coupled on-line with HPLC. The selectivity of method for the extraction of acesulfame was evaluated in the presence of some sweets (saccharine, caffeine, and aspartame) in the beverage sample. This robust tool showed proper selectivity toward acesulfame and was used for fifty extractions without any noticeable obstruction.

## 6. Other Novel Methods for Preparation of MSG

**Figure 6:**
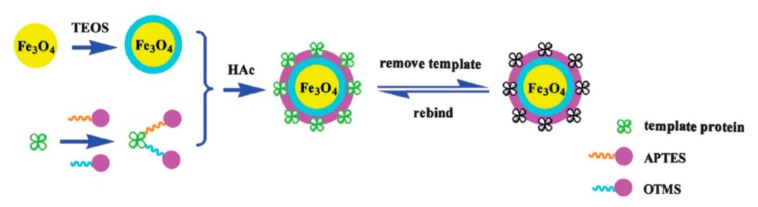
Nano-magnetic MIP preparation with sol-gel [47].

Recently some interesting methods for MSG preparation have been developed which we will discuss here briefly. In a novel study a type of uniform nanomagnetic MIP sorbent was prepared by the sol-gel methodology and applied for the recognition of bovine serum albumin (BSA) [47]. The Fe_3_O_4_@BSA–MIPs showed 5 nm size, which can facilitate the mass transfer, and a high saturation magnetization (43.82 emu g^−1^), which allowed it to be easily separated from solution using an external magnetic field. These nanomaterials showed a proper equilibrium time (15 min) with good imprinting factor and selectivity coefficient (16.4 and 4.65). Fe_3_O_4_@BSA–MIPs was used successfully for the separation and enrichment of BSA from a bovine blood sample with good recovery and stability. In this method TEOS, APTES and octyltrimethoxysilane (OTMS) as monomer and crosslinkers were used in two-step process to create core–shell nano-MSG on a Fe_3_O_4_@SiO_2_ surface. The template molecule, APTES and OTMS were mixed separately and were then added to the mixture of Fe_3_O_4_ and TEOS. The process was followed by adding acid and finally template molecules were removed to generate imprinted cavities. The preparation process of Fe_3_O_4_@BSA–MIPs is shown in Figure 6.

In an interesting approach a MIP based on an ionic liquid (IL) sorbent on the surface of multiwall carbon nanotubes (MWCNTs) was prepared utilizing sol-gel methodology [48]. In this method 3-aminopropyltriethoxysilane-modified multiwall carbon nanotube (MWCNT-APTES) was used as support surface, BSA as the template, an alkoxy-functionalized IL (1-(3-trimethoxysilylpropyl)-3-methyl imidazolium chloride, [TMSPMIM]Cl) was both the functional monomer and the sol-gel catalyst, and TEOS as the crosslinking agent were used. In this process, MWCNTs@BSA-MIPILs were modified with APTES and a mixture of template-monomer was added to ensure proper covalent binding. The next step was accomplished with TEOS for hydrolysis and polycondensation. Finally, the elimination of the template from the matrix revealed the specific binding spots. In this controllable method effective parameters to increase the selectivity (MIP cavity shapes) and decrease non-specific binding such as pH value, ionic strength of the incubated were addressed and optimized.

Recently, a novel monolithic magnetic molecularly imprinted nanoparticle stir-bar was prepared with sol-gel methodology for the extraction of thiabendazole (TBZ) and carbendazim (CBZ) from orange samples [49]. In this method, oleic acid was used to modify the surface and then a sol-gel procedure was employed to encapsulate the particles. The sol-gel method can help overcome some weaknessed of previous common MIP preparation techniques. However sol-gel techniques cannot solve all issues, and to prove this statement a study was performed by the Kadhirvel group [50]. In this work MIPs were packed in column in both acrylic and sol-gel tridimensional networks for selective extraction of naproxen. All related parameters were optimized, and the results showed sol-gel improve the selectivity, but the acrylic approach presented better mass transfer, efficiency and porosity. This interesting approach opens a window to show the importance of preparing composite imprinted materials by mixing acrylic and silica-based precursors in future studies.

In a further approach microspheric particles of MIX was prepared by filling up the pores of spherical, mesoporous, bare silica particles with a pregelification mixture utilizing pressure. Then a thin layer of MIX was created on the mesopore using gelification and a drying step. In order to prevent extensive outer-surface deposition several parameters needed to be optimized such as the amount of porogen, pressurization time and the selection of a proper washing solvent for the pore-filling step. The results proved that uniform pore-filled silica particles increased the adsorption capacity and facilitated the analyte binding process. This spherical composite showed high selectivity for the separation of (*S*)-naproxen in the presence of ibuprofen (α = 4.9, imprinting factor = 13). In comparison with bulk polymerization methods this methoid displayed outstanding column efficiency (9 vs. 1.2 theoretical plates/cm) [51]. 

MSG is a flexible method and it has also been applied in real-life. In such a real-life application, MSG was prepared and used as an antidandruff agent [52]. To prepare a MSG composite a mixture of silane, tetra(C_1_-C_4_)alkyl orthosilicate, porogen solvent and C_14_-C_20_ fatty acid (as template molecule) were used. The results proved that MSG can selectivity trap the C_14_-C_20_ fatty acid, the mean reason for dandruff formation. Additionally, in an attractive work a water-compatible MSG polymer was used for the controlled release of salicylic acid as an anti-inflammatory drug [53]. In vivo investigations showed that MSG has lower binding capacity and higher imprinting factor in water media in comparison with organic solvents. Moreover, from safety and toxicity aspects MSG particles of more than 300 nm in size would not cross the skin barrier. MIPs still are not prevalent for drug delivery applications due to their poor compatibility in polar environments and the results of referred work proved that MSG could be a potential alternative in this field. As a conclusion, the recent applications of MSG in different sample preparation fields are summarized in Table 1.

## 7. Conclusions

In this review recent applications of sol-gel methodology for the preparation of imprinted polymers were discussed. Sol-gel methodology not only can facilitate MIP preparation but also improve the thermal and chemical satability. MSG materiala can be prepared in various formats and can be applied easily in diffrent sample peparation techniques. However some aspects of the MSG method need improvement, such as better selectivity, sensitivity and longer lifetime to be more applicable in the near future.

## Figures and Tables

**Table 1:** MSG application in diffenert sample prepartion methods.

Analyte	Sample Preparation Method	Instrumentation	Matrix	Ref
Enrofloxacin	SPE	HPLC	Fish and chicken samples	[22]
Methyl-3-quinoxaline-2-carboxylic acid and quinoxaline-2-carboxylic acid	SPE	HPLC	Pork muscle	[23]
Cloxacilloic acid	SPE	HPLC	Cloxacillin	[24]
2,4-Dichlorophenoxyacetic	SPE	FT-IR	Aqueous media	[25]
Florfenicol	SPE	HPLC	Meat samples	[26]
Chrysoidine	SPE	HPLC	Food samples	[27]
Vitamin D3	SPE	HPLC	Aqueous samples	[28]
Polar organophosphorus pesticides	SPE	LC-MS	Almond oil	[29]
Phthalate esters	SPE	GC-MS	Water sample	[30]
Sulfonicacid dyes	SPE	HPLC	Beverage samples	[31]
Sudan IV	SPE	HPLC	Chili samples	[32]
Cu(II)	SPE	HPLC	Herbal medicines	[33]
Bisphenol A	SPE	HPLC	Aqueous samples	[34]
Iprodione fungicide	SPE	HPLC	Wine	[35]
Methadone	SPE	HPLC	Human plasma	[36]
Diethylstilbestrol	SPE	HPLC	Milk samples	[37]
Bisphenol A	SPE	HPLC	Beverage samples	[38]
Fentanyl	Capillary-SPME	HPLC	Urine and plasma samples	[39]
Organophosphorous pesticides	Fiber-SPME	GC	Vegetable samples	[40]
Bilirubin	Needle-SMPE	LC-MS/MS	Plasma and urine samples	[41]
Organophosphorous pesticides	Stainless steel wire-SPME	GC	Fresh and dry foods	[42]
l-Tyrosine	Monolithic in tip	LC-MS/MS	Human plasma and urine samples	[43]
Vanillin and methyl vanillin	Monolithic in tip	HPLC	Milk powder	[44]
Hippuric acid	Hollow fiber liquid-phase microextraction	LC-MS/MS	Human plasma and urine samples	[45]
Acesulfame	Nanofiber-SPME	HPLC	Beverage samples	[46]
Bovine serum albumin	Magnetic nanomaterials	FT-IR	Bovine blood sample	[47]
Bovine serum albumin	Ionic liquid/Multiwall carbon nanotube	UV-Vis	Human serum albumin and bovine hemoglobin	[48]
Thiabendazole and carbendazim	Sorptive monolith nanoparticles/stir-bar	HPLC	Orange samples	[49]
Naproxen	Xerogel	HPLC	Aqueous samples	[50]
(*S*)-Naproxen	Xerogel composite	HPLC	Aqueous samples	[51]
C_14_-C_20_ fatty acid	Xerogel composite	Fluorescence spectroscopy	Hair samples	[52]
Salicylic acid	Xerogel composite	Fluorescence spectroscopy	Skin *in vivo*	[53]
